# Facilitating Charge Reactions in Al‐S Batteries with Redox Mediators

**DOI:** 10.1002/cssc.202100973

**Published:** 2021-06-23

**Authors:** He Li, John Lampkin, Nuria Garcia‐Araez

**Affiliations:** ^1^ Chemistry University of Southampton University Road Southampton SO17 1BJ United Kingdom

**Keywords:** aluminium-sulfur batteries, batteries, deep eutectic solvents, energy storage, redox mediators

## Abstract

The Al−S battery is a promising next‐generation battery candidate due to high abundance of both aluminium and sulfur. However, the sluggish kinetics of the Al−S battery reactions produces very high overpotentials. Here, for the first time, it was demonstrated that the incorporation of redox mediators could dramatically improve the kinetics of Al−S batteries. On the example of iodide redox mediators, it was shown that the charging voltage of Al−S batteries could be decreased by about 0.23 V with as little as 2.3 wt% of redox mediator added as electrolyte additive. Control electrochemical measurements, without prior discharge of the battery, demonstrated that >97 % of the charge capacity was due to the desired oxidation of Al_2_S_3_ and polysulfides, and X‐ray diffraction experiments confirmed the formation of sulfur as the final charge product. The beneficial role of redox mediators was demonstrated with cheap and environmentally friendly electrolytes made of urea and AlCl_3_. This work showed that dramatic performance improvements could be achieved with low concentration of electrolyte additives, and therefore, much further performance improvements could be sought by combining multiple additives.

## Graphical abstract

**Faster kinetics with mediator**: Sluggish reaction kinetics is one major factor holding back Al−S battery development, since it leads to a large voltage hysteresis and low energy efficiency. However, here the incorporation of redox mediators is shown to dramatically improve the reaction kinetics and, thus, decrease reaction overpotentials. With as low as 2.3 wt% of iodide mediator, the charge voltage of Al−S batteries is decreased by about 0.23 V, using a cheap and environmentally friendly electrolyte. These results show that the use of mediators is a promising new approach to unlock the potential of Al−S batteries.

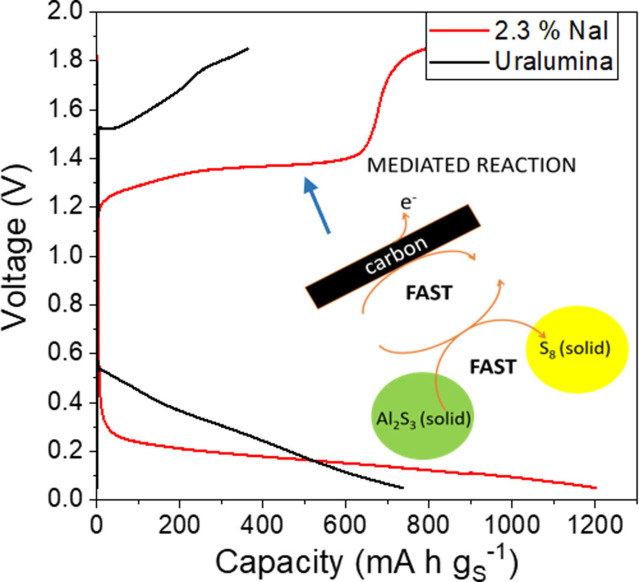

## Introduction

The increasing global population, and ever‐growing demand of energy, has generated a spectacular growth in the Li‐ion market in recent years, and much more accelerated growth is expected in coming years. However, the limited abundance of lithium on earth raises questions about the capacity of Li‐ion batteries to fulfil the vastly increasing demand of energy storage, and thus researchers are eagerly exploring alternative battery technologies like Na‐ion batteries, Mg‐ion batteries, Al‐ion batteries and others.^[1]^

Among the beyond Li‐ion battery candidates, the Al−S battery can play a very important role in the future since aluminium is the most abundant metal element in the Earth's crust (8.23 %) and can be easily manufactured. When applied as anode material, multivalent aluminium has a very high theoretical gravimetric and volumetric capacity (2980 Ah kg^−1^ and 8040 Ah L^−1^, respectively).[^1d^, ^2^] Coupling an aluminium anode with a sulfur cathode, another abundant material, forms a Al−S battery with a theoretical voltage of approximately 1.23 V, high theoretical specific energy (1319 Wh kg^−1^) and high theoretical energy density (2981 Wh L^−1^).^[3]^

Up to now, only a few research articles about Al−S batteries have been published (see details in Table S1).^[4]^ The reported values of specific capacity are promising, and the capacity retention is also improving gradually. These early works show that Al−S batteries have great potential to become a high‐energy‐density and low‐cost battery. However, most of these studies applied a low current density and employed thin cathodes with low sulfur content. Improvements in the thickness and sulfur content of the cathode are required to make a commercially viable Al−S battery. This is illustrated in Table S1 by calculating the specific discharge energy of the battery, normalised by the mass of the cathode and anode, showing that the low sulfur content in the cathodes results in a significant energy penalty.^[4k]^ Another significant problem of the state‐of‐the‐art Al−S batteries is their very high charging voltages, which is addressed in this work by the use of redox mediators. Here we demonstrate that redox mediators can be successfully applied to Al−S batteries, made with sulfur electrodes with commercially relevant sulfur content (60 wt%), to significantly decrease the charging voltage by 0.23 V.

A high charging voltage in Al−S batteries is highly problematic because it can promote degradation reactions and the corrosion of current collectors.^[5]^ But equally damaging is the effect of a high charging voltage on the battery round trip efficiency (where the round trip efficiency is defined as the ratio of discharge energy to charge energy). For applications such as grid storage, a high round trip efficiency is critical; otherwise, a large fraction of the energy generated is wasted, instead of getting stored in the battery, and in addition the wasted energy is released as heat, which, in turn, causes further degradation issues.[^5a^, ^6^]

Here we propose a novel strategy to decrease the charging voltage of Al−S batteries: the introduction of redox mediators as electrolyte additives to catalyse the kinetics of the charge reaction. Although redox mediators have been used in Li−O_2_,^[7]^ Na−O_2_ ^[8]^ and Li−S^[9]^ batteries, this is the first time that they are incorporated in an Al−S battery. A significant decrease in the charging voltage of 0.23 V is obtained for all of the electrolytes here studied. In addition to the conventional ionic liquid used in most previous works on Al−S batteries (a mixture of [EMIM]Cl, 1‐ethyl‐3‐methylimidazolium chloride, and AlCl_3_), here we also investigate the use of deep eutectic electrolytes made with urea, which have significant advantages in terms of cost and biodegradability.^[10]^

There are only two previous studies on Al−S using electrolyte additives. Yu et al. showed that the incorporation of a lithium salt [LiCF_3_SO_3_ or lithium bis(trifluoromethanesulfonyl)imide (LiTFSI)] in a concentration of 0.5 m (equivalent to 6–11 wt%) in the [EMIM]Cl‐AlCl_3_ ionic liquid produced a drastic improvement in the cycle life of Al−S batteries.^[4d]^ Yang et al. showed that the use of an [EMIM]Br‐AlCl_3_ ionic liquid, instead of [EMIM]Cl‐AlCl_3_, produced a drastic improvement in the rate capability of Al−S batteries, which was ascribed to the catalytic effect of Al_2_Cl_6_Br^−^ species, present in the electrolyte at a concentration close to 29 wt%.^[4e]^ In this work, we investigate four redox mediator candidates (NaBr, LiBr, NaI and LiI) at a low concentration (<3 wt%) and we demonstrate that the redox reactions undergone by I^−^/I_3_^−^ species are highly effective at mediating the charge reaction of an Al−S cell. It is also worth mentioning that several electrolyte additives can be combined to obtain further improvements in performance, as it is commonly done for Li‐ion batteries.^[11]^ Therefore, the results shown here open the door to many further studies on the use of electrolyte additives in the development of Al−S batteries.

## Results and Discussion

**Figure 1:**
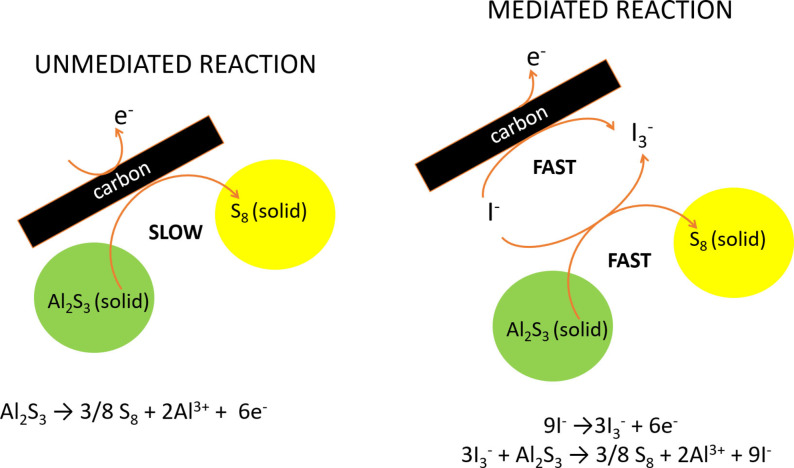
Proposed mechanism of non‐mediated and mediated (with iodide) charging reactions of Al−S batteries. Note that, in the reactions, Al^3+^ is used to refer to the Al‐containing species present in the electrolyte (e. g., AlCl_4_^−^, Al_2_Cl_7_^−^, etc.)

The focus of this work is the investigation of the use of redox mediators to facilitate the charging reaction, with the specific purpose of decreasing the charging voltage of Al−S batteries. The mechanism of operation of redox mediators for charging Al−S batteries is illustrated in Figure 1, on the example of iodide as redox mediator, since we found that the addition of NaI or LiI as electrolyte additive produces a drastic decrease in the charging voltage.

While the oxidation of Al_2_S_3_ in the battery without redox mediators has slow kinetics (thus, requires high overpotentials) [Eq. 1]:

(1) $$\mathrm{Al}{}_{2}S{}_{3}\to 3/8\;S{}_{8}+2\mathrm{Al}{}^{3+}+6e{}^{-}$$

where Al^3+^ is used to refer to the Al‐containing species present in the electrolyte (e. g., AlCl_4_^−^, Al_2_Cl_7_^−^, etc.). In the presence of the iodide redox mediator, the charge reaction is fast, because it proceeds via the following two fast steps [Eqs. (2) and 3]:

(2) $$9I{}^{-}\to 3I{}_{3}{}^{-}+6e{}^{-}$$

(3) $$\mathrm{Al}{}_{2}S{}_{3}+3I{}_{3}{}^{-}\to 3/8\;S{}_{8}+2\mathrm{Al}{}^{3+}+9I{}^{-}$$

And since iodide is regenerated in Eq. (3), it can then undergo further oxidation reactions [Eq. (2)], and consequently the sequence of Eqs. (2) and (3) can take place many times producing a very fast overall reaction. Note that the combination of Eqs. (2) and (3) is simply the oxidation of Al_2_S_3_ to sulfur [Eq. (1)], but since the reaction pathway is changed, the rate of the reaction is enhanced when it occurs via Eqs. (2) and (3). Consequently, in the proposed reaction scheme, iodide acts as a catalyst, since it is involved in the reaction of Al_2_S_3_ oxidation, thus enhancing the kinetics, but it is not consumed in the net, overall reaction.

The faster kinetics of the reaction mediated by iodide could be due to the fact that the oxidation of Al_2_S_3_ takes place as a chemical reaction with the tri‐iodide oxidising species [Eq. (3)]. On the contrary, without mediators [Eq. (1)], Al_2_S_3_ (which is an insulating solid) has to transfer the electrons to the current collector, via the carbon conductive additive. The slow kinetics of the charge reaction of Al−S batteries is not seen in Li−S batteries, because in the later, polysulfides facilitate the charging reaction.^[12]^ However, in the electrolytes used in Al−S batteries, the solubility of polysulfides is much smaller[^4a^, ^4c^] and, consequently, the charge reaction kinetics is sluggish.

Four redox mediators were investigated in this work: NaBr, LiBr, NaI and LiI. The use of halide salts has the advantage that halides do not undergo any reductive degradation reaction at the potential of aluminium deposition. Indeed, NaI has been used as additive in aluminium electrodeposition from molten salts.^[13]^ The use of both bromide and iodide salts enables the study of the effect of the redox potential of the mediator, since iodide oxidises at a much lower potential than bromide. Finally, the use of lithium and sodium salts enabled the investigation of whether the nature of the cation produced a significant effect in the mediation of the battery reactions.

The effect of redox mediators on the performance of Al−S batteries was first investigated in the Uralumina electrolyte (made with urea and AlCl_3_ mixed in a 1 : 1.5 molar ratio), since its low cost makes is particularly promising for Al−S batteries. Two series of electrolyte formulations of Uralumina with redox mediators were investigated (see Table 1). In the first series, the mediator partially replaced AlCl_3_, and in the second one, the mediator partially replaced urea.

**Table 1:** Concentration of mediators (given in wt % of total solution) in the Uralumina electrolytes.^[a]^

Series 1 of mediators in Uralumina				Series 2 of mediators in Uralumina	
LiBr	NaBr	LiI	NaI	LiI	NaI
0.8	0.8	0.8	0.8	0.7	0.7
2.3	2.3	2.3	2.3	–	–
–	–	3.9	3.9	–	–
–	–	7.7	7.7	–	–

[a] Series 1 and 2 were prepared with the mediator partially replacing AlCl_3_ or urea, respectively, in the base Uralumina formulation (urea/AlCl_3_ 1 : 1.5 molar ratio). The molar concentrations are reported in Table S2.

**Figure 2:**
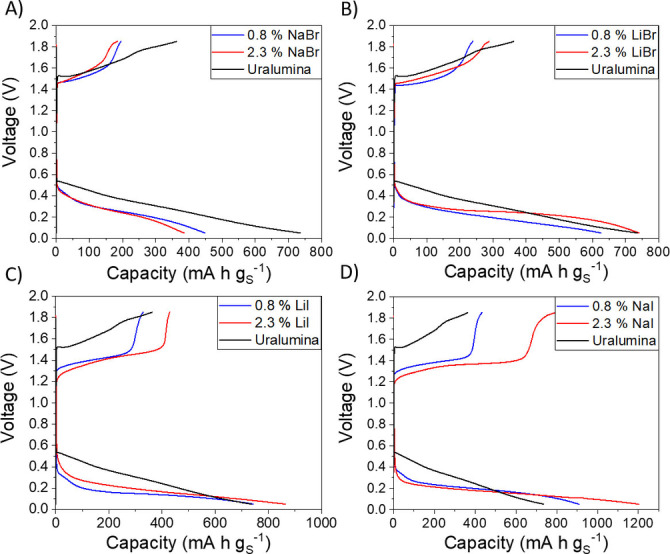
Voltage profiles of unmediated and mediated Al−S cells in the first discharge/charge cycle with Uralumina electrolyte. The electrolytes were prepared by partially replacing the AlCl_3_ in the base Uralumina electrolyte (series 1, see Table 1). Experiments used a specific current of 50 mA g_S_^−1^ and the voltage range is 0.05–1.85 V. The sulfur loading in the electrodes was 1.3–1.9 mg cm^−2^.

**Figure 3:**
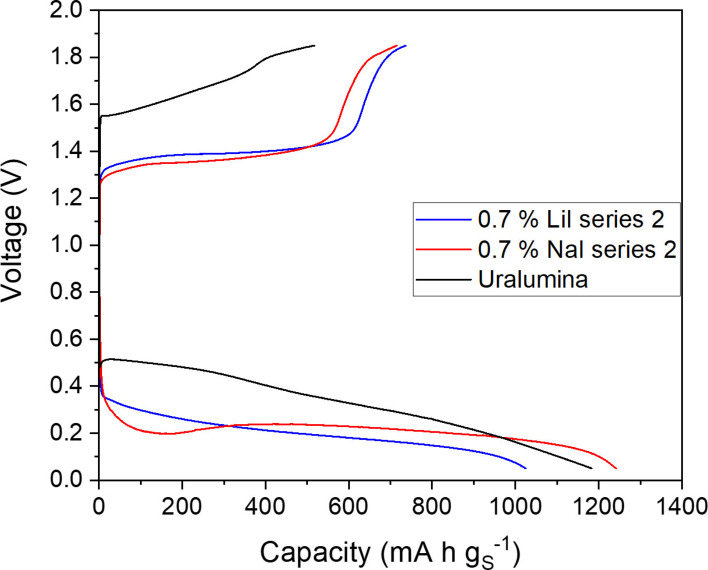
As in Figure 2 but with electrolytes prepared by partially replacing the urea in the base Uralumina by the mediator (series 2, Table 1). The sulfur loading in the electrodes was 1–1.1 mg cm^−2^. The differences in capacity with respect to the results in Figure 2 are due to the difference in sulfur loading, as shown in Figure 5.

Figures 2 and 3 compare the voltage profiles of Al−S batteries with Uralumina electrolytes with and without the selected mediators (NaBr, LiBr, NaI and LiI). It is clear that introducing the iodide mediators, even in small concentrations (between 0.7 and 2.3 wt%), produces a very dramatic decrease in the charge voltage. On the contrary, the voltage profiles with bromide mediators are similar to those in the base Uralumina without mediators. In both cases, using lithium or sodium salts produces very similar results, suggesting that the role of the cation in the reactions is only minor. Some differences in the capacity values can be observed, but they are related to the different thickness of the electrodes, as discussed below in more detail. For all the cells, the discharge capacity is higher than the charge capacity; this is due to electrolyte degradation reactions that contribute to the first discharge capacity, as discussed in previous work.^[4k]^

**Figure 4:**
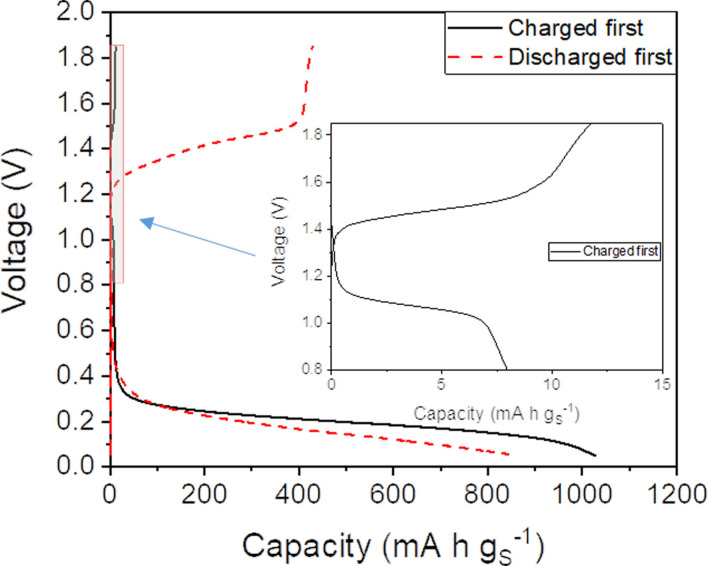
Voltage profile of Al−S cells with Uralumina electrolyte with 2.3 wt% LiI mediator (series 1, see Table 1), obtained by charging the cells before discharge (solid black curves, inset shows a zoom‐in). The voltage profile obtained with the standard protocol, with discharge prior to charge, is also shown for comparison (dashed red curves). All conditions as in Figure 2. The sulfur loading of charged first is 1.36 mg cm^−2^ and discharge first is 1.48 mg cm^−2^.

In order to verify the presence of a true mediation of the charge reaction, it is necessary to study the reaction of the mediator itself. Our working hypothesis is that the charge capacity is largely due to the reactions of oxidation of Al_2_S_3_ and polysulfides that are formed in the previous discharge of the battery. Consequently, if the cell was charged without a previous discharge, then the capacity will be very small. Following this reasoning, we performed experiments in which an Al−S was assembled and then charged to 1.85 V without a prior discharge to 0.05 V (see results in Figure 4, solid black curves). Since the cell contained an Uralumina electrolyte with LiI mediator, the charge capacity is due to the reaction of iodide to tri‐iodide [Eq. (2)], and since the iodide concentration is very small, the capacity is also very small (≈12 mAh g_S_^−1^). On the other hand, when the cell is discharged prior to charge (Figure 4, dashed red curves), then the charge capacity is much bigger (≈429 mAh g_S_^−1^). The difference in charge capacities shows that only around 2.8 % of the charge capacity is due to electrochemical reactions undergone by the mediator alone, whereas the remaining 97.2 % are Al_2_S_3_ and polysulfide reactions (whose kinetics are enhanced, in the present case, by the presence of the mediator). In conclusion, the electrochemical reactions of the mediator alone involve very small capacities, and thus produce very little effect on the voltage profiles in Figures 2 and 3.

**Figure 5:**
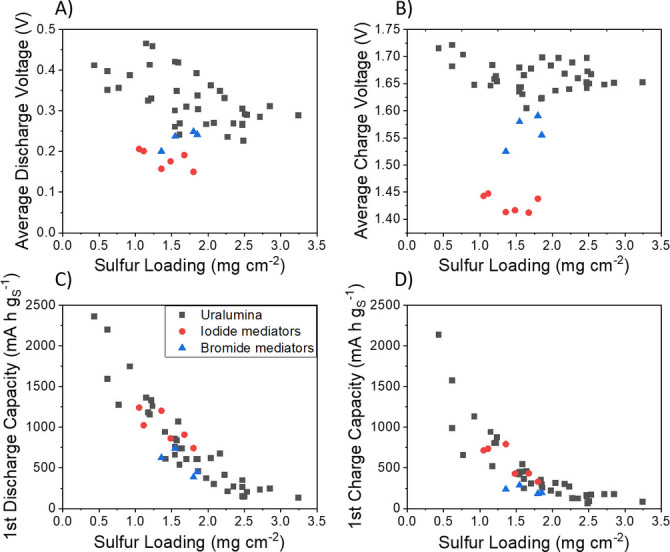
Comparison of key performance parameters of Al−S batteries in Uralumina electrolyte with and without redox mediators, for the first discharge and charge cycle. The black points are the results with the bare Uralumina, and the red and blue points are with iodide or bromide mediators, respectively. The average discharge and charge voltage values are calculated using Equation (S2).

A quantitative analysis of the key performance parameters (first discharge and charge capacities and average discharge and charge voltages) of the Al−S cells with and without mediators is shown in Figure 5. In previous work, we systematically varied the sulfur loading in the cathodes by varying the cathode thickness, while keeping the same cathode formulation, and we observed a systematic decrease in the specific discharge capacity (normalised by the mass of sulfur) of Al−S cells in the base Uralumina electrolyte as the sulfur loading (and thus, the electrode thickness) increased.^[4k]^ In Figure 5 we combine the results obtained in the base Uralumina electrolyte, reported previously, with the new results with redox mediators, and for the sake of clarity, the results with redox mediators are grouped into iodide (red circles) and bromide (blue triangles) mediators. Full information of the nature of the mediator in each of these cells is provided in Figure S2.

Figure 5 shows that the presence of mediator does not produce a significant change in the discharge or charge capacities. This is as expected, since the mediator is designed to facilitate the charge reaction (Al_2_S_3_ oxidation), but not to affect the amount of Al_2_S_3_ that is formed on discharge. As shown in previous work, the decrease in specific discharge capacity as the cathode thickness increases is due to the slow transport of species within the highly viscous Uralumina electrolyte, and consequently, with thick cathodes, only the region of the electrode that is closer to the separator (which then can be accessed fast enough by the transport of electrolyte species) participates in the electrochemical reactions.^[4k]^ Alternative approaches need to be explored to enhance the discharge capacity with thick electrodes, which could involve the synergistic combination of new cathode formulations with low tortuosity, electrolyte additives to reduce the viscosity of the electrolyte and redox mediators designed to facilitate the discharge reaction.

Importantly, Figure 5 also shows that the addition of iodide mediators produces a marked decrease (≈0.23 V) of the average charge voltage, from 1.66±0.03 V without mediators to 1.43±0.02 V with iodide mediators. The decrease in charge voltage is highly advantageous, since it means that less energy has to be applied to the battery to charge it. Unfortunately, the addition of iodide also produces a decrease, albeit small (≈0.15 V), of the average discharge voltage, from 0.33±0.03 V without mediators to 0.18±0.02 V with iodide mediators. The decrease in the discharge voltage is disadvantageous, since it means that the battery will deliver less energy during discharge. However, the decrease in discharge voltage observed in the presence of mediators is probably caused by the increase in electrolyte viscosity that is produced by the mediators in the Uralumina electrolyte. Further work is required to develop alternative approaches to overcome such mass transport limitation (e. g., non‐tortuous electrodes, redox mediators for discharge etc.), and then, the electrolyte viscosity will no longer affect the discharge process, and consequently, the mediators will also not affect the discharge process. Figure S4 shows the long‐term cycling stability of the batteries, which, unfortunately, is not improved by the presence of the mediators, and it is another area that requires further improvement.

In view of the success of the incorporation of iodide mediators to decrease the charge voltage of Al−S cells, further experiments were performed with higher concentrations of mediators (3.9 and 7.7 wt%, Figure S3). Unfortunately, it was observed that the specific discharge capacities were small, most likely due to the very significant increase of the electrolyte viscosity obtained at high mediator concentrations. As discussed above, due to mass transport limitations with highly viscous electrolytes, very thin electrodes are required in order achieve full sulfur utilisation.^[4k]^ Consequently, the voltage profiles are much more affected by the reactions of the mediator alone, since the sulfur utilisation is low and the concentrations of mediators are high. Figure S3 shows that, in the second discharge, a plateau at around 1.0 V appears, which can be ascribed to the reduction of tri‐iodide species (formed in the previous charge) to iodide. Indeed, the inset in Figure 4, obtained with a smaller concentration of iodide mediator, also shows the presence of a small voltage plateau at around 1.0 V, although with much smaller associated capacity. In all cases, iodide is oxidised to tri‐iodide on charge (at ≈1.4 V), and then tri‐iodide is reduced to iodide on discharge (at ≈1.0 V). In the experiments shown in Figure 4, since the initial concentration of iodide was smaller, the amount of tri‐iodide generated on charge was also smaller, and thus, the smaller capacity of the process of tri‐iodide reduction to iodide at around 1.0 V.

Comparing the behaviour of redox mediators at different concentrations (Figures 2, 3 and S3), it is seen that concentrations of mediator of 1 and 2.3 wt% are close to optimal, since they produce the beneficial effect of the decrease in the charge voltage but do not produce a noticeable decrease in discharge capacity due to the increased electrolyte viscosity. Consequently, further experiments were then performed with this selected mediator concentrations with different electrolytes, with the purpose of investigating whether the mediation action of iodide was generally applicable to any electrolyte for Al−S batteries. Two ionic‐liquid electrolytes were selected for this study: [EMIM]Cl‐AlCl_3_ and [BMP]Cl‐AlCl_3_ (1‐butyl‐1‐methylpyrrolidinium chloride), both of which have been used before for Al−S batteries.[^4a^, ^4e^] These ionic liquids exhibit much lower viscosity than the Uralumina electrolyte (see Table 2), and thus the discharge reactions are less affected by mass transport limitations.

**Table 2:** Density and viscosity of base electrolytes.

Electrolyte	Density [g mL^−1^]	Viscosity [mPa s]
Uralumina	1.564	218
[EMIM]Cl‐AlCl_3_	1.360	13
[BMP]Cl‐AlCl_3_	1.349	62

**Figure 6:**
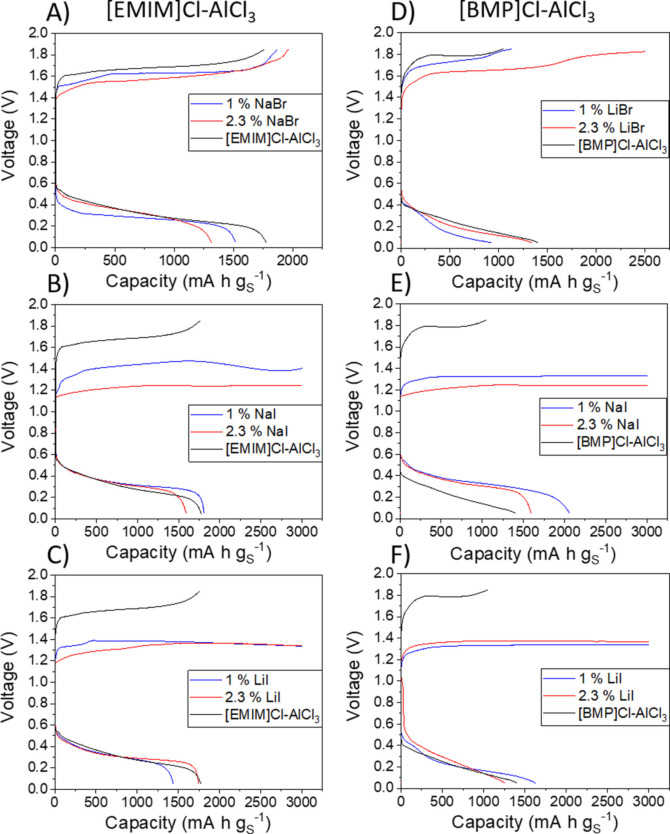
Voltage profiles of unmediated and mediated Al−S cells in the first discharge/charge cycle with [EMIM]Cl‐AlCl_3_ (A–C) and [BMP]Cl‐AlCl_3_ (D–F) electrolytes. Experiments used a specific current of 50 mA g_S_^−1^ and the voltage range is 0.05–1.85 V. The sulfur loading in the electrodes was 0.9–1.4 mg cm^−2^ for [EMIM]Cl‐containing cells and 0.9–1.6 mg cm^−2^ for [BMP]Cl‐containing cells.

Figure 6 shows the results of the incorporation of iodide and bromide redox mediators in Al−S batteries with [EMIM]Cl‐AlCl_3_ and [BMP]Cl‐AlCl_3_ electrolytes. The concentrations of mediators studied are listed in Table 3. Figure 6 shows that bromide mediators produce very little effect in the electrochemical behaviour of the batteries, whereas iodide mediators produce a drastic decrease in the charging voltage.

**Table 3:** Concentration of mediators (given in wt % of total solution) in [EMIM]Cl‐AlCl_3_ (in a 1 : 1.5 molar ratio) and [BMP]Cl‐AlCl_3_ (in a 1 : 1.5 molar ratio) electrolytes. The molar concentrations are reported in Table S3.

Mediators in [EMIM]Cl‐AlCl_3_			Mediators in [BMP]Cl‐AlCl_3_		
NaBr	LiI	NaI	LiBr	LiI	NaI
1.0	1.0	1.0	1.0	1.0	1.0
2.3	2.3	2.3	2.3	2.3	2.3

The quantitative analysis of the key performance parameters (discharge and charge capacities and average discharge and charge average voltage), compared to the behaviour in the bare [EMIM]Cl‐AlCl_3_ electrolyte reported in our previous work,^[4k]^ is shown in Figure S6. It is seen that the discharge performance parameters (discharge capacity and voltage) are not affected by the presence of mediators, as expected. On the other hand, the presence of iodide mediators reduces the charging voltage by approximately 0.28 V (the average discharge voltage in the bare [EMIM]Cl‐AlCl_3_ electrolyte is 1.61±0.05 V, whereas with iodide mediators it is 1.33±0.08 V). Therefore, the beneficial effect of iodide in decreasing the charging voltage of Al−S batteries takes place in the [EMIM]Cl‐AlCl_3_ and [BMP]Cl‐AlCl_3_ electrolytes, in a similar way as in the Uralumina electrolyte. Unfortunately, in the [EMIM]Cl‐AlCl_3_ and [BMP]Cl‐AlCl_3_ electrolytes, the presence of iodide mediators also produces an overcharge issue, since the selected upper charging voltage limit of 1.85 V was not achieved in the cells with iodide mediator even after 60 h of charging (corresponding to a charge capacity limit of 3000 mA h g_S_^−1^).

The problem of overcharge in the presence of iodide redox mediators can be explained by the shuttling of iodide and tri‐iodide species between the aluminium and sulfur electrodes (see sketch in Figure S7). Since the mass transport rate of species becomes faster with less viscous electrolytes, the problem of overcharge is seen in [EMIM]Cl‐AlCl_3_ and [BMP]Cl‐AlCl_3_ electrolytes but not in the Uralumina electrolyte, since the latter is much more viscous. This hypothesis is confirmed by the fact that when the cells with an [EMIM]Cl‐AlCl_3_ electrolyte with iodide mediator are charged without a prior discharge (Figure S8), a significant overcharge is seen, which must be ascribed to redox reactions of the mediator alone (i. e., the oxidation of iodide to tri‐iodide, which involves a high capacity due to the shuttling mechanism). Figure S8 also shows that the cells with mediator that are charged without prior discharge exhibit lower charging voltage that cells that are charged after the initial discharge. These differences can be explained by the fact that the formation of Al_2_S_3_ during discharge produces passivation of the electrode. However, the most important finding is that cells overcharge in both cases, thus showing that the reaction of the mediator by itself, without involvement of Al_2_S_3_, has associated a very large capacity, due to the mediator shuttling. In contrast, Figure 4 shows that, in the Uralumina electrolyte, the iodide redox reactions are minimal, since very low capacities are observed in the case when the cells are not discharged prior to the charging process.

In conclusion, the use of iodide redox mediator is only suitable for Al−S batteries with high viscosity electrolytes. However, for commercial applications, the development of Al−S batteries with polymer gel electrolytes is particularly promising, since their solid nature prevents the leaks of the toxic liquid electrolyte outside the cell and confers additional protection against moisture.^[14]^ For such application with polymer gel electrolytes, the problem of overcharge would be eliminated due to the slower rate of mass transport, and thus the implementation of redox mediators is ideally suited.

**Figure 7:**
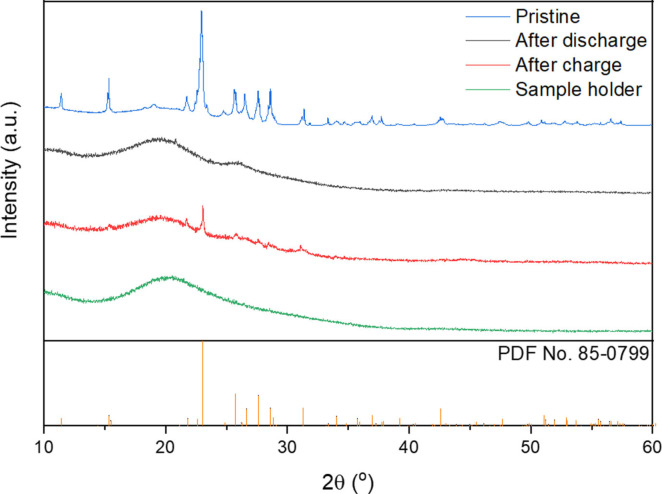
XRD pattern (grazing incidence, incident angle of 5°) of a sulfur cathode in the pristine form (blue), after discharge (black), and after charge (red) in an Al−S cell with 2.3 wt% NaBr in [EMIM]Cl‐AlCl_3_ electrolyte, using a specific current of 50 mA g^−1^. The pattern obtained with the XRD sample holder is also included (green). The sulfur peak positions are below (yellow) PDF No. 85–0799.

Finally, in order to obtain further proof of the reaction mechanism, the sulfur cathodes of Al−S batteries, after discharge and charge, were characterised by X‐ray diffraction (XRD). Figure 7 shows the XRD patterns of the pristine sulfur cathode and after discharge and charge in an Al−S cell with 2.3 wt% NaBr in [EMIM]Cl‐AlCl_3_ electrolyte. The pristine sulfur cathode XRD pattern contains the characteristic peaks of crystalline sulfur, as reported previously.[^4a^, ^4b^] Then, after discharge, the peaks disappear, demonstrating that all the sulfur in the electrode has been consumed in electrochemical reactions, as also reported previously.[^4a^, ^4b^] Lastly, after charge, the peaks characteristic of crystalline sulfur reappear.^[15]^ The reappearance of sulfur after charge is very significant and confirms the proposed reaction mechanism of sulfur reduction to Al_2_S_3_ on discharge, and subsequent oxidation of Al_2_S_3_ to sulfur on charge. Although Al_2_S_3_ is not detected, possibly because it is formed in amorphous form, the reappearance of sulfur on charge demonstrates that the electrochemical reactions are reversible.

## Conclusion

This work demonstrates, for the first time, the implementation of redox mediators to facilitate the reactions of Al−S batteries. Redox mediators are redox active species that catalyse an electrochemical reaction by enabling an alternative pathway, involving the reversible oxidation and reduction of the redox mediator, in such a way that the redox mediator is not consumed in the reaction.

Consequently, low concentrations of redox mediators can produce a dramatic effect on the reaction kinetics and, indeed, here we show that the incorporation of LiI or NaI at a low concentration of only 2.3 wt% produces a dramatic decrease in the charging voltage of Al−S batteries containing a low‐cost urea‐based electrolyte. In order to verify that the charge reaction was not simply due to the oxidation of the iodide redox mediator, additional electrochemical measurements were performed with cells that had not been discharged prior to charge. In this way, we could quantify the capacity due to the reaction of the iodide redox mediator alone, which was found to be <3 % of the total capacity. In addition, X‐ray diffraction characterisation of the sulfur electrodes after discharge and charge in Al−S cells demonstrated the consumption of sulfur after discharge and reformation of sulfur after charge, thus providing further evidence of the proposed reaction mechanism.

Unfortunately, the incorporation of the iodide redox mediators in less viscous electrolytes, made with imidazolium or pyrrolidinium based ionic liquid, produced overcharge problems due to shutting of the redox mediator between the sulfur and aluminium electrodes. Consequently, the redox mediators here discovered are only suitable for highly viscous electrolytes, such as polymer gel electrolytes, which, on the other hand, are the most suitable option for safe and reliable Al−S batteries.

## Experimental Section

### Cathode preparation

The procedure of sulfur electrode preparation is the same as reported previously.^[4k]^ A mixture of 180 mg sulfur (Aldrich, 100‐mesh) and 90 mg multi‐walled carbon nanotubes (CNT, 98 % basis, Sigma‐Aldrich) was prepared (sulfur/carbon=2 : 1 *w*/*w*) and mixed using a pestle and mortar, and then it was transferred into a 23 mL polytetrafluoroethylene (PTFE)‐lined autoclave (Parr Instrument Company). A pre‐heated oven (Genlab Ltd. MINO/6) was used to heat the autoclave at 155 °C for 6.5 h to promote the impregnation of sulfur into the carbon. Once cooled to room temperature, the product was collected. A binder solution was prepared using 24 mg polyethylene oxide (PEO, *M*_w_=600000, Sigma‐Aldrich) and 12 mg polyvinylpyrrolidone (PVP, Aldrich), which were dissolved into acetonitrile (1.2 mL, HiPerSolv, ACS) and ethanol (0.6 mL, Fisher, ≥99.8 % analytical grade) under a fume hood. The sulfur and carbon nanotube mixture, after impregnation, was added to the binder solution along with a magnetic stirring bar and stirred for no less than 2 h. The resulting ink had a formula composition of 58.8 : 29.4 : 7.9 : 3.9 (S/CNT/PEO/PVP, wt %), for simplicity, this will be referred to as 60 wt% sulfur. The ink was then coated onto a pre‐cut and cleaned piece of Mo foil (25 μm thickness, ≥99.9 % trace metals basis, Sigma‐Aldrich) using a doctor blade. Once the solvents had evaporated, the coated sheet was cut into round discs (Ø=11 mm, Hohsen hand‐held precision punch). The discs were dried under vacuum at room temperature on a Büchi line for 2 days. Each electrode was weighed before every experiment (OHAUS Adventurer^TM^, AR0640) inside an Ar‐filled glovebox (O_2_ and H_2_O <5 ppm) to obtain the precise weight of the active material.

### Electrolyte preparation

[EMIM]Cl‐AlCl_3_ (1 : 1.5 molar ratio) was purchased from Sigma‐Aldrich. Uralumina (urea/AlCl_3_=1 : 1.5 molar ratio) and [BMP]Cl‐AlCl_3_ (1 : 1.5 molar ratio) were generously provided by Dr. Christopher Zaleski (Scionix Ltd.), Dr. Igor Efimov and Prof. Karl Ryder (University of Leicester). The electrolytes were used as received and were stored in an Ar‐filled glovebox.

The electrolytes containing mediator were prepared in two ways. In the first procedure, the mediator (measured by wt %) was dissolved into the electrolyte directly. This procedure was chosen to study mediators in [EMIM]Cl‐AlCl_3_ and [BMP]Cl‐AlCl_3_ electrolytes. In the second procedure, different electrolytes were synthesized by reaction of the base reagents in the presence of a selected concentration of mediator. This procedure was used to study the mediators in the Uralumina electrolyte, and the different electrolytes were preparing by partially substituting one of the base reagents of Uralumina (urea or AlCl_3_) by the mediator. The first series of Uralumina electrolytes with mediator were prepared by substituting 1, 3, 5 or 10 wt% of AlCl_3_ by the mediator. The second series were obtained by substituting 3 wt% of urea by the mediator. The mediators chosen were sodium iodide (NaI, anhydrous, 99.999 %, Sigma‐Aldrich), lithium iodide (LiI, anhydrous, 99.99 %, Sigma‐Aldrich), sodium bromide (NaBr, 99.99 %, Sigma‐Aldrich) and lithium bromide (LiBr, anhydrous, 99.999 %, Sigma‐Aldrich).

The preparation of the Uralumina electrolytes with mediators was done inside the glovebox. The chosen mediators, AlCl_3_ (99.99 %, Sigma‐Aldrich) and Urea (99 %, Sigma‐Aldrich), were individually ground with a pestle and mortar and then transferred into a Duran bottle. A glass rod was used to stir the reaction mixture quickly. The reagents started to react quickly and formed a solution in an exothermic reaction. The solution was stirred quickly for a few minutes after the initial reaction to facilitate the reaction of all the reagents. The solution was left to cool down overnight and it was then filtered using a pipette filter (Whatman® GD/X syringe filters, 1 μm pore size).

### Cell construction

The electrochemical cells and cell construction procedure are the same as reported previously.^[4k]^ All the cell components are shown in Figure S1. Aluminium Swagelok unions were used as the cell body, since aluminium is stable in contact with the electrolyte (unless polarised to oxidation potentials, which was avoided by assuring electrical disconnection between the sulfur and aluminium electrodes and the cell body). The inside of the cell body was lined with Mylar film and the cells were sealed using perfluoroalkoxy alkanes (PFA) Swagelok ferrules, which prevent the electrodes from short‐circuiting with the cell body and produce an air‐tight seal. 11 mm diameter discs of high‐purity aluminium (0.2 mm thick, Puratronic, 99.997 % metals basis) were used as anode. The cathodes were 11 mm diameter discs of ink‐coated sulfur composite on Mo foil (see section 2.1 for details). The separators used were 12 mm discs of Whatman GF/F glass microfiber filters with 120 μL of electrolyte pipetted onto them. The current collectors were cylindrical bars made from aluminium (purity ≥97.5 %) and tungsten (purity ≥99 %) for the anode and cathode, respectively.

Prior to cell assembly, all Swagelok cell components were sonicated in ethanol (Fisher, ≥99.8 % analytical grade) and then dried in an oven (Genlab E3, E3DWC100/N) at 80 °C overnight. Additionally, the current collector bars and caps were polished using silicon carbide paper (1200 grit, 3 M) and ethanol, this is to remove the thick oxidation layer and any deeper contamination from the surface. Once dry, the cells were partially constructed to test for short‐circuiting with a multimeter. They were then immediately transferred into an Ar‐filled glovebox and assembled in the glovebox.

### Electrochemical testing and characterisation

Electrochemical testing was carried out using a VMP3 potentiostat (BioLogic Science Instruments) with the cells placed in a Memmert climatic chamber set to 25 °C. Galvanostatic cycling with potential limitation (GCPL) experiments were done applying a lower and upper voltage limit of 0.05 and 1.85 V, respectively. The applied specific current (normalized to the mass of sulfur) was 50 mA g_S_^−1^. Cells were left at rest at the open circuit potential for 6 h, and then they were discharged (to 0.05 V) and charged (to 1.85 V), for a total of 10 cycles. In some cases, cells were charged prior to discharge in order to study the oxidation of the mediator.

XRD patterns were recorded with a Rigaku Smartlab diffractometer (Rigaku Corporation) using CuK_α_ radiation operated at 45 kV, 150 mA. Samples were placed in a special X‐ray transparent dome shaped sample holder for air‐sensitive materials purchased from Bruker (Ø=55.5 mm PMMA disc with Ø=25 mm×1 mm specimen well). A piece of glass was used as a flat surface to elevate the electrode during measurements.

The viscosity of the electrolytes was measured using a Cannon‐Fenske viscometer tube (Sigma‐Aldrich) and the experiment was recorded using a mobile phone camera (Samsung Galaxy S10+). The viscosity values were calculated from the times the electrolytes required to flow between two marks in the viscometer, as estimated from the video [see Eq. (S3)].

## Conflict of interest

The authors declare no conflict of interest.

## Supporting information

As a service to our authors and readers, this journal provides supporting information supplied by the authors. Such materials are peer reviewed and may be re‐organized for online delivery, but are not copy‐edited or typeset. Technical support issues arising from supporting information (other than missing files) should be addressed to the authors.

Supporting Information
