# Supplementary material for: Facilitating Charge Reactions in Al‐S Batteries with Redox Mediators
Source: ChemSusChem. 2021 Jun 23;14(15):3139–46. doi: 10.1002/cssc.202100973 (PMC8453840; doi:10.1002/cssc.202100973)
Supplement: Supplementary file 1 — Supporting Information [file CSSC-14-3139-s001.pdf]

ChemSusChem

Supporting Information

Facilitating Charge Reactions in Al-S Batteries with Redox Mediators

He Li[+], John Lampkin[+], and Nuria Garcia-Araez*© 2021 The Authors. ChemSusChem published by Wiley-VCH GmbH. This is an open access article under the terms of the Creative Commons Attribution License, which permits use, distribution and reproduction in any medium, provided the original work is properly cited.

Table S1. Comparison of the main electrochemical results reported in Al-S battery studies, in chronological order (from older to newer)

Work	Electrode Composition	Electrolyte	Sulfur Content (wt.%)	Sulfur Loading (mg cm^{-2})	Specific Current (mA g_S^{-1})	Initial Specific Discharge Capacity (mAh g_S^{-1})	Charge Voltage (V)[a]	Discharge Voltage (V)[a]	Specific Energy of Initial Discharge (Wh $kg_{electrodes}^{-1}$)[b]
G. Cohn et. al, 2015[1]	S, Ketjen black, PVDF (50:30:20) on stainless steel (**non-rechargeable**)	[EMIM]Cl-$AlCl_3$ (1:1.5)	50	1.1	30	1400	n/a	1.2	656
T. Gao et. al, 2016[[2]]	S on activated carbon cloth	[EMIM]Cl-$AlCl_3$ (1:1.3)	n/a	0.8-1.0	50	1320	1.4	0.65	n/a
X. Yu et. al, 2017[[3]]	Spreading the mixture of S and ionic liquid electrolyte onto activated CNF paper (S:CNF≈1:2)	[EMIM]Cl-$AlCl_3$ (1:1.3)	33	~1.0	~30 (C/50)	1350	~1.75	1.05	395
X. Yu et. al, 2018[[4]]	Spreading the mixture of S and ionic liquid electrolyte onto activated CNF paper (S:CNF≈1:2)	0.5 M $LiCF_3SO_3$ in [EMIM]Cl-$AlCl_3$ (1:1.25)	33	~1.0	~30 (C/50)	1250	~1.55	0.76	265
H. Yang et. al, 2018[[5]]	S, CMK-3, Ketjen black, PTFE (40:40:10:10) on glassy carbon	[EMIM]Br-$AlCl_3$ (1:1.3)	40	n/a	251	1500	~1.3	~0.5	245
		[NBMP]Br-$AlCl_3$ (1:1.3)	40	n/a	251	1390	~1.6	~0.5	227
Y. Bian et. al, 2018[[6]]	S, MWCNT, polyacrylic latex (10:80:10) on Ni foil	Urea-$AlCl_3$ (1:1.4)	10	0.42	1000	740	~2.2	~1.7	119
W. Wang et. al, 2018[[7]]	10 % S. SPAN, Ketjenblack and PTFE (80:10:10) on carbon paper.	[EMIM]Cl-$AlCl_3$ (1:1.5)	10	0.12	25	320	~2.2	0.3	9
W. Chu et. al, 2019[[8]]	S, CMK-3 1:1, with 10 % PTFE and 10 % Super C on Mo foil	Acetamide- $AlCl_3$ (1:1.3)	40	0.25	100	2100	~1.4	0.55	377
K. Zhang et. al, 2019[[9]]	BN/S/C (6:1:2) with 10 % PVDF coated on Pt coated OHP organic film	[EMIM]Cl-$AlCl_3$ (1:1.3)	10	~0.3	100	~275	~2.2	1.15	~30
X. Zheng et. Al, 2020[[10]]	Reduced Graphene Oxide (rGO)/ CoS_2 mixture with Super P and PVDF (80:10:10) on Ta foil	[EMIM]Cl-$AlCl_3$ (1:1.3)	60	0.2-0.4	50	1127[e]	~1.5	~0.2[e]	~101
J. Lampkin et. al, 2020[11]	S, CNT with PEO and PVP coated on Mo foil (58.8:29.4:7.9:3.9)	[EMIM]Cl-$AlCl_3$ (1:1.5)	58.8	~0.4-3.5	50	1404[c]	1.6[d]	0.31[d]	192
		Acetamide-$AlCl_3$ (1:1.5)	58.8	~0.4-3.5	50	2129[c]	1.6[d]	0.42[d]	395
		Urea-$AlCl_3$ (1:1.5)	58.8	~0.4-3.5	50	2359[c]	1.66[d]	0.41[d]	428

[a] The discharge and charge voltages are estimated as equal to the discharge/charge plateaus in the voltage vs capacity plot, and when the values were obtained from the graph, the symbol ~ has been added. [b] The specific energy of the initial discharge is normalized to the total mass of the sulfur and aluminium electrodes (see equation S1). [c] The thinnest mass loading (~0.4 mg cm^{-2}) is used when reporting the specific capacity and specific energy. [d] This work reports the average values of discharge and charge voltage (see equation S2). [e] This work also uses rGO separators to produce an increase in discharge voltage and capacity after 10 cycles.

Equation S1. The specific energy of the initial discharge (normalized to the total mass of the sulfur and aluminium electrodes) was calculated from:

Specific energy of initial discharge = $V_{DISCH} * Q_{DISCH}/(100/wt.\% + 0.561)$

where:

V_{DISCH} = average discharge voltage

Q_{DISCH} = total specific discharge capacity

$wt.\%$ = mass % of sulfur in the sulfur electrode

This calculation includes the mass of the aluminium electrode required to react with the sulfur electrode to produce Al_2S_3. This calculation takes into account that each gram of sulfur in the sulfur electrode requires a mass of 0.561 grams of aluminium for the formation of Al_2S_3, and that each gram of sulfur in the sulfur electrode is associated with a total mass of the sulfur electrode of 100/wt.% grams, where wt.% is the mass percentage of sulfur in the sulfur electrode.

An analogous equation can be used to calculate the specific energy of the initial charge (normalized to the total mass of the sulfur and aluminium electrodes):

Specific energy of initial charge = $V_{CHARG} * Q_{CHARG}/(100/wt.\% + 0.561)$

where:

V_{CHARG} = average charge voltage

Q_{CHARG} = total specific charge capacity

Equation S2. The average discharge voltage, V_{DISCH}, was calculated using the following equation:

$$V_{DISCH} = \frac{\int_0^{Q_{DISCH}} V dQ}{Q_{DISCH}}$$

where:

V: discharge voltage

Q = specific capacity

Q_{DISCH} = total specific discharge capacity (at the lower cut off voltage)

And an analogous equation was used for the average charge voltage V_{CHARG}:

$$V_{CHARG} = \frac{\int_0^{Q_{CHARG}} V dQ}{Q_{CHARG}}$$

where:

V: charge voltage

Q = specific capacity

Q_{CHARG} = total specific charge capacity (at the upper cut off voltage)

Equation S3. The dynamic viscosity of the electrolyte, η, was calculated from:

$$\eta = K * t * \rho$$

where *K* is the viscometer constant (*K*= 0.1 cSt/s), *t* is the time taken for the electrolyte to freely flow between two marks in the viscometer and ρ is density of the electrolyte.

Table S2. Estimated molar concentration of mediators (given in moles per litre of solution) in the Uralumina electrolytes solutions. Series 1 and 2 were prepared with the mediator partially replacing $AlCl_3$ or urea, respectively, in the base Uralumina formulation (urea: $AlCl_3$ 1:1.5 molar ratio). The molar concentrations are calculated from the weight percentages of mediator in the solutions (Table 1), considering that the densities of the solutions equal that of the base Uralumina (1.564 g mL^{-1}).

**Series 1 of mediators in Uralumina**				**Series 2 of mediators in Uralumina**	
LiBr	NaBr	LiI	NaI	LiI	NaI
0.14	0.12	0.09	0.08	0.08	0.07
0.41	0.35	0.27	0.24		
		0.46	0.41		
		0.90	0.80		

Table S3. Estimated molar concentration of mediators dissolved in [EMIM]Cl-$AlCl_3$ and in [BMP]Cl-$AlCl_3$ electrolytes. The molar concentrations are calculated from the weight percentages of mediator in the solutions (Table 2), considering that the densities of the solutions equal that of the base electrolytes (1.360 g mL^{-1} for [EMIM]Cl-$AlCl_3$ and 1.349 g mL^{-1} for [BMP]Cl-$AlCl_3$).

**Mediators in [EMIM]Cl-$AlCl_3$**			**Mediators in [BMP]Cl-$AlCl_3$**		
NaBr	LiI	NaI	LiBr	LiI	NaI
0.13	0.10	0.09	0.16	0.10	0.09
0.30	0.23	0.21	0.36	0.23	0.21

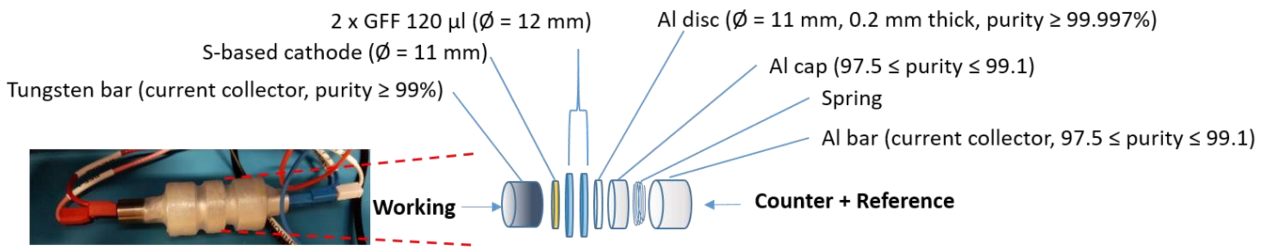

Figure S1. Photo and illustration of the cell components used for Al-S cell cycling.

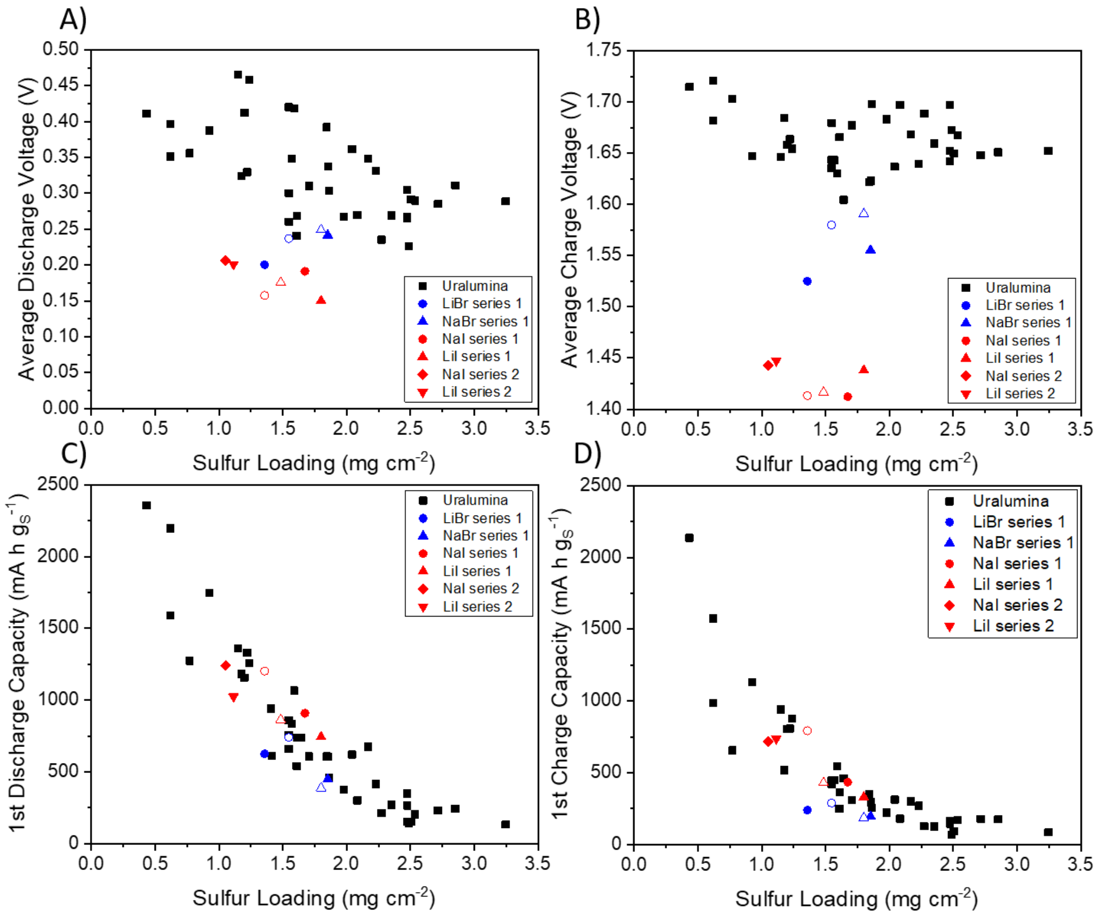

Figure S2. Comparison of key performance parameters of Al-S batteries in Uralumina electrolyte with and without redox mediators. For series 1 data points, filled symbols are cells with 0.8 wt.% of mediators and non-filled symbols are 2.3 wt.% of mediator.

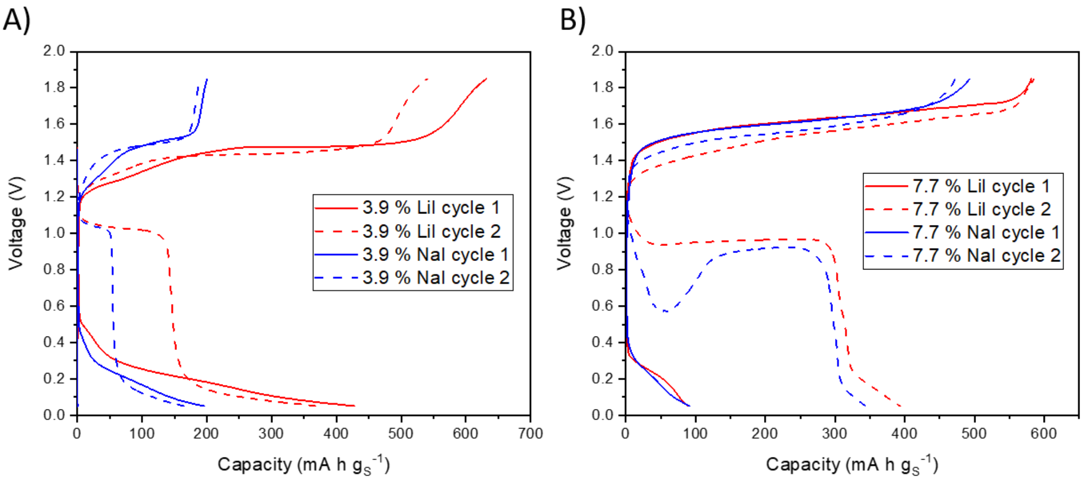

Figure S3. Voltage profiles of Al-S cells with Uralumina electrolyte with different concentrations of redox mediators, as indicated, in the first (solid lines) and second (dashed lines) discharge/charge cycles. The electrolytes were prepared by partially replacing the $AlCl_3$ in the base Uralumina electrolyte (series 1, see table 1). Experiments used a specific current of 50 mA g_S^{-1} and the voltage range is 0.05-1.85 V. The sulfur loading in the electrodes was 1.3-1.8 mg cm^{-2}.

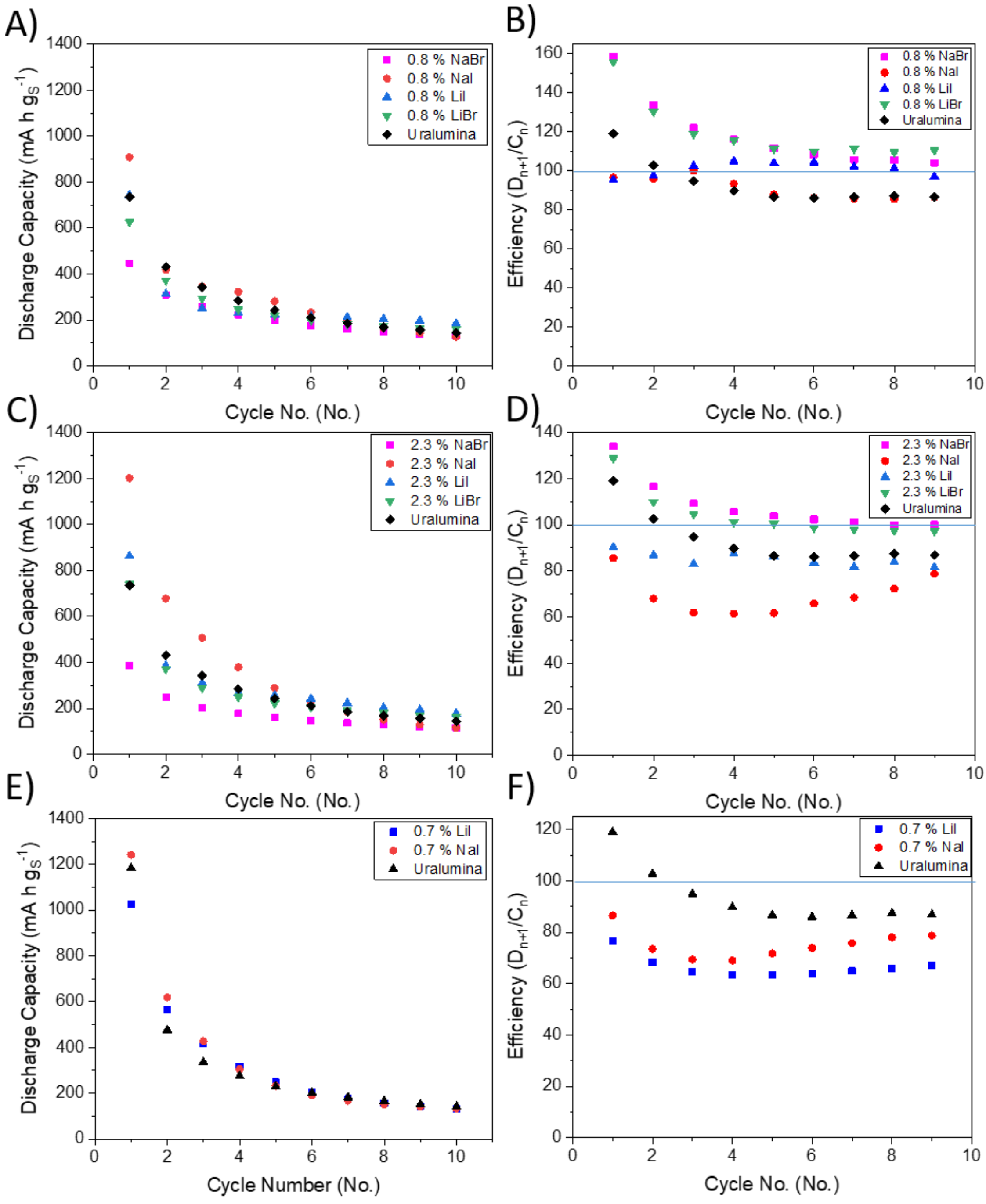

Figure S4. Evolution of the discharge capacity with cycle number (left) and Efficiency with cycle number (right) for Al-S cells with Uralumina electrolyte with different concentrations of redox mediators: A+B) 0.8 % and C+D) 2.3 % mediator (series 1, mediator partially replacing $AlCl_3$), and E+F) 0.7 % mediator (series 2, mediator partially replacing urea). The sulfur loading in the electrodes was 1.3-1.9 mg cm^{-2} (Figure S4A&B), 1.3-1.8 mg cm^{-2} (Figure S4C&D) and 1.0-1.7 mg cm^{-2} (Figure S4E&F) respectively.

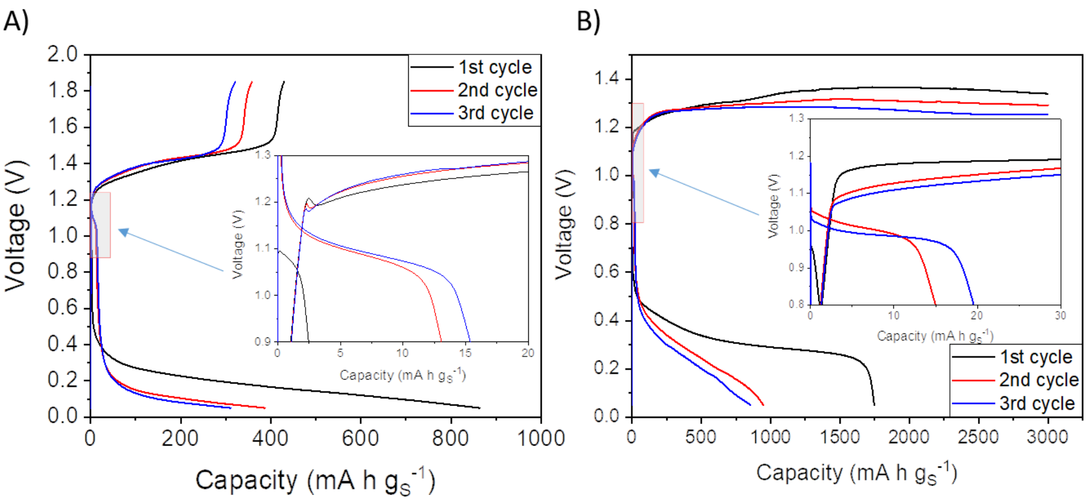

Figure S5. Voltage profiles of Al-S cells in the first three cycles in A) Uralumina with 2.3 wt.% LiI and B) [EMIM]Cl-$AlCl_3$ with of 2.3 wt.% LiI, showing an enlarged inset image displaying the additional voltage plateau that appears on the second discharge. Experiment used a specific current of 50 mA g_S^{-1} and the voltage range is 0.05-1.85 V. The sulfur loading in the electrodes was: A) 1.48 and B) 0.99 mg cm^{-2}.

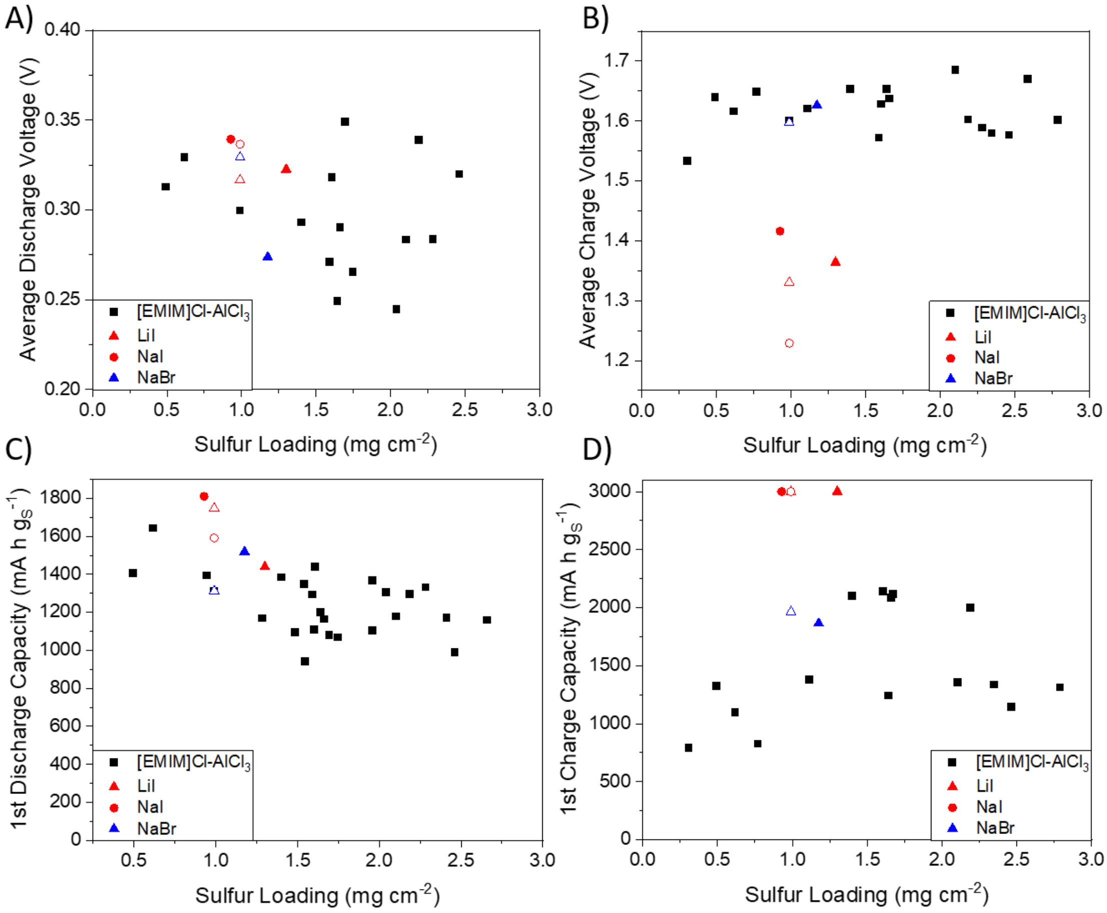

Figure S6. Comparison of key performance parameters of Al-S batteries in [EMIM]Cl-$AlCl_3$ electrolyte with and without redox mediators. 1 and 2.3 wt.% mediator solutions correspond to filled and non-filled data points respectively. In the charge capacity plot, the cells with Iodide redox mediator exceeded the time 60 hour cut-off limit, and hence the capacity values plotted equal the capacity limit of 3000 mAh g^{-1} set in the measurements.

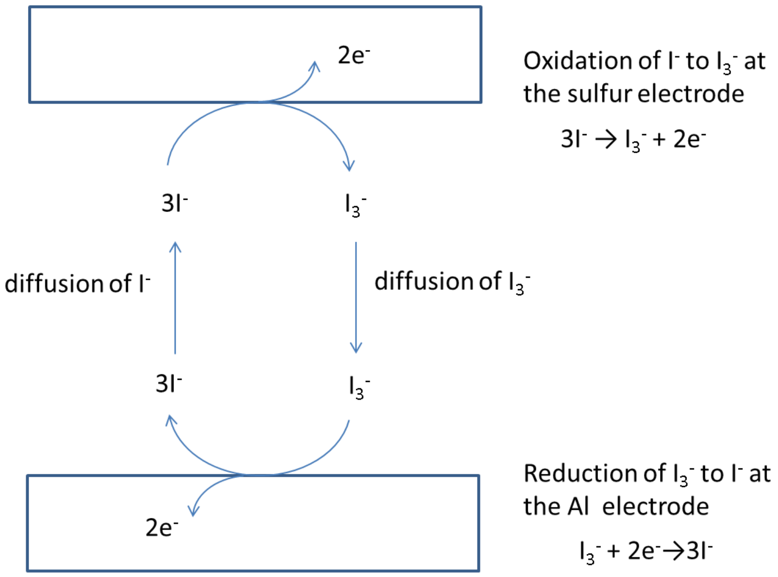

Figure S7. Illustration of the mechanism of iodide and tri-iodide shuttling in Al-S batteries, producing overcharge.

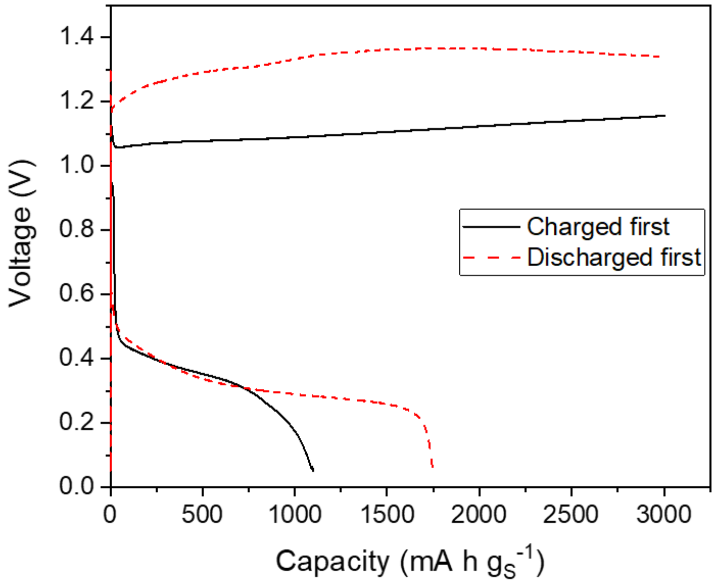

Figure S8. Voltage profile of Al-S cells with [EMIM]Cl-$AlCl_3$ electrolyte with 2.3 wt.% LiI mediator (series 1, see Table 1), obtained by charging the cells before discharge (solid black curves, inset shows a zoom-in). The voltage profile obtained with the standard protocol, with discharge prior to charge, is also shown for comparison (dashed red curves). Experiments used a specific current of 50 mA g_S^{-1} and the voltage range is 0.05-1.85 V. The sulfur loading of charged first is 1.11 mg cm^{-2} and discharge first is 0.99 mg cm^{-2}.

References

[1] G. Cohn, L. Ma, L. A. Archer, *Journal of Power Sources* **2015**, *283*, 416-422.

[2] T. Gao, X. Li, X. Wang, J. Hu, F. Han, X. Fan, L. Suo, A. J. Pearse, S. B. Lee, G. W. Rubloff, K. J. Gaskell, M. Noked, C. Wang, *Angew Chem Int Ed Engl* **2016**, *55*, 9898-9901.

[3] X. W. Yu, A. Manthiram, *Advanced Energy Materials* **2017**, *7*.

[4] X. W. Yu, M. J. Boyer, G. S. Hwang, A. Manthiram, *Chem* **2018**, *4*, 586-598.

[5] H. Yang, L. Yin, J. Liang, Z. Sun, Y. Wang, H. Li, K. He, L. Ma, Z. Peng, S. Qiu, C. Sun, H. M. Cheng, F. Li, *Angew Chem Int Ed Engl* **2018**, *57*, 1898-1902.

[6] Y. H. Bian, Y. Li, Z. C. Yu, H. Chen, K. W. Du, C. C. Qiu, G. X. Zhang, Z. C. Lv, M. C. Lin, *Chemelectrochem* **2018**, *5*, 3607-3611.

[7] W. X. Wang, Z. Cao, G. A. Elia, Y. Q. Wu, W. Wahyudi, E. Abou-Hamad, A. H. Emwas, L. Cavallo, L. J. Li, J. Ming, *Acs Energy Letters* **2018**, *3*, 2899-2907.

[8] W. Q. Chu, X. Zhang, J. Wang, S. Zhao, S. Q. Liu, H. Yu, *Energy Storage Materials* **2019**, *22*, 418-423.

[9] K. Zhang, T. H. Lee, J. H. Cha, R. S. Varma, J. W. Choi, H. W. Jang, M. Shokouhimehr, *Sci Rep* **2019**, *9*, 13573.

[10] X. Zheng, R. Tang, Y. Zhang, L. Ma, X. Wang, Y. Dong, G. Kong, L. Wei, *Sustainable Energy & Fuels* **2020**, *4*, 1630-1641.

[11] J. Lampkin, H. Li, L. Furness, R. Raccichini, N. Garcia-Araez, *ChemSusChem* **2020**, *13*, 3514-3523.
